# Acute tetrahydrobiopterin supplementation attenuates sympathetic vasoconstrictor responsiveness in resting and contracting skeletal muscle of healthy rats

**DOI:** 10.14814/phy2.12164

**Published:** 2014-10-15

**Authors:** Nicholas G. Jendzjowsky, Timothy P. Just, Kelvin E. Jones, Darren S. DeLorey

**Affiliations:** 1 Faculty of Physical Education and Recreation, University of Alberta, Edmonton, Alberta, Canada

**Keywords:** Exercise, nitric oxide, sympathetic nervous system, sympatholysis, vascular conductance

## Abstract

Tetrahydrobiopterin (BH_4_) is an essential cofactor for the production of nitric oxide (NO) and supplementation with BH_4_ improves NO‐dependent vasodilation. NO also reduces sympathetic vasoconstrictor responsiveness in resting and contracting skeletal muscle. Thus, we hypothesized that supplementation with BH_4_ would blunt sympathetic vasoconstrictor responsiveness in resting and contracting skeletal muscle. Sprague‐Dawley rats (*n* = 15, 399 ± 57 g) were anesthetized and instrumented with an indwelling brachial artery catheter, femoral artery flow probe, and a stimulating electrode on the lumbar sympathetic chain. Triceps surae muscles were stimulated to contract rhythmically at 30% and 60% of maximal contractile force (MCF). The percentage change of femoral vascular conductance (%FVC) in response to sympathetic stimulations delivered at 2 and 5 Hz was determined at rest and during muscle contraction in control and acute BH_4_ supplementation (20 mg·kg^−1^ + 10 mg·kg^−1^·h^−1^, IA) conditions. BH_4_ reduced (*P* < 0.05) the vasoconstrictor response to sympathetic stimulation (i.e., decrease in FVC) at rest (Control: 2 Hz: −28 ± 5%FVC; 5 Hz: −45 ± 5%; BH_4_: 2 Hz: −17 ± 4%FVC; 5 Hz: −34 ± 7%FVC) and during muscular contraction at 30% MCF (Control: 2 Hz: −14 ± 6%FVC; 5 Hz: −28 ± 11%; BH_4_: 2 Hz: −6 ± 6%FVC; 5 Hz: −16 ± 10%) and 60% MCF (Control: 2 Hz: −7 ± 3%FVC; 5 Hz: −16 ± 6%FVC; BH_4_: 2 Hz: −2 ± 3%FVC; 5 Hz: −11 ± 6%FVC). These data are consistent with our hypothesis that acute BH_4_ supplementation decreases sympathetic vasoconstrictor responsiveness in resting and contracting skeletal muscle.

## Abstract (precis)

Tetrahydrobiopterin (BH4) is an essential cofactor for the production of nitric oxide (NO) and NO reduces sympathetic vasoconstrictor responsiveness in the skeletal muscle vascular bed. Thus, we hypothesized that supplementation with BH4 would blunt sympathetic vasoconstrictor responsiveness. The data demonstrate that acute BH4 supplementation decreases sympathetic vasoconstrictor responsiveness in resting and contracting skeletal muscle.

## Introduction

Skeletal muscle blood flow is regulated by a balance between sympathetic nervous system mediated vasoconstriction and local vasodilation (Saltin et al. 1998). Substances released from muscle tissue and/or the vascular endothelium can blunt sympathetic vasoconstriction and the inhibition of sympathetic vasoconstriction is an important mechanism for the matching of skeletal muscle blood flow to metabolic demand (DeLorey et al. 2002; VanTeeffelen and Segal 2003; Thomas and Segal 2004; Bagher and Segal 2011). Indeed, a decline in the ability to blunt sympathetic vasoconstriction may increase vascular resistance and contribute to skeletal muscle hypoperfusion (Saltin and Mortensen 2012). Thus, interventions that improve the blunting of sympathetic vasoconstriction may improve the control of skeletal muscle vascular conductance.

The mechanism(s) responsible for the blunting of sympathetic vasoconstriction has not been definitively established; however, removal of the endothelium or pharmacological blockade of nitric oxide (NO) production enhances the constrictor response to sympathetic stimulation in resting and contracting skeletal muscle, demonstrating that NO inhibits vasoconstriction (Ohyanagi et al. 1992; Habler et al. 1997; Thomas and Victor 1998; Thomas et al. 1998, 2003; Sander et al. 2000; Jendzjowsky and DeLorey 2013b, 2013c). Our laboratory recently demonstrated that exercise training improved the inhibition of sympathetic vasoconstriction in resting and contracting muscle through a NO‐dependent mechanism (Jendzjowsky and DeLorey 2013c).

Tetrahydrobiopterin (BH_4_) is an essential cofactor required for NO production by the NO synthase (NOS) group of enzymes (Katusic et al. 2009; Forstermann 2010; Forstermann and Sessa 2012). Decreased BH_4_ availability has been associated with reduced NO‐dependent vascular function (Bevers et al. 2006; Forstermann 2010; Forstermann and Sessa 2012; Santhanam et al. 2012). In contrast, supplementation with exogenous BH_4_ has been shown to improve NO‐dependent vasodilation in healthy individuals and to restore ACh and flow‐mediated vasodilation in populations with endothelial dysfunction (Higashi et al. 2002; Eskurza et al. 2005; Cosentino et al. 2008).

Exogenous BH_4_ treatment may also augment NO‐mediated inhibition of sympathetic vasoconstriction in the skeletal muscle vasculature. However, to date, studies of the effects of acute BH_4_ supplementation on skeletal muscle vascular control have focused on the effects of BH_4_ on endothelium‐dependent vasodilation (Gruhn et al. 2001; Higashi et al. 2002; Delp et al. 2008; Sindler et al. 2009). Whether acute BH_4_ supplementation can enhance the inhibition of sympathetic vasoconstriction in resting and contracting skeletal muscle has not been investigated.

Therefore, the purpose of this study was to determine the effect of acute BH_4_ supplementation on sympathetic vasoconstrictor responsiveness in resting and contracting skeletal muscle of healthy rats. It was hypothesized that acute BH_4_ supplementation would reduce sympathetic vasoconstrictor responsiveness in resting and contracting skeletal muscle.

## Methods

### Animals and animal care

Male Sprague‐Dawley rats (*n* = 15; 399 ± 57 g) were obtained from the institutional breeding colony. Rats were housed in pairs in a 12:12‐h light–dark cycle, environmentally controlled (22–24°C, 40–70% humidity) room. Water and rat chow (Lab Diet 5001, PMI Nutrition, Brentwood, MO) were provided ad libitum. All experiments were conducted in accordance with the Canadian Council on Animal Care Guidelines and Policies with approval from the Animal Care and Use Committee: Health Sciences for the University of Alberta.

### Instrumentation

Anesthesia was induced by inhalation of isoflurane (3–3.5%, balance O_2_). During isoflurane anesthesia, the right jugular vein was cannulated and anesthesia was subsequently maintained by infusion of *α*‐chloralose (8–16 mg·kg^−1^·h^−1^) and urethane (50–100 mg·kg^−1^·h^−1^). The depth of anesthesia was assessed by the stability of arterial blood pressure, heart rate (HR), and the absence of a withdrawal reflex in response to a painful stimulus (i.e., paw‐pinch). A tracheotomy was performed to allow spontaneous breathing of room air. We have previously demonstrated that arterial blood gases and acid base status are well maintained at rest and during muscle contraction in this preparation (Jendzjowsky and DeLorey 2013c). Thus, arterial blood gases were checked periodically in these experiments to confirm the maintenance of blood gas and acid base status. Arterial blood pressure was measured by a pressure transducer (Abbott, North Chicago, IL) that was attached to a cannula implanted in the left brachial artery. HR was derived from the blood pressure waveform and mean arterial pressure (MAP) was calculated. The left femoral artery and vein were cannulated for the delivery of solutions and blood sampling. Blood flow was measured using a transit‐time flow probe (0.7 V Transonic Systems, Ithaca, NY) placed around the right femoral artery and connected to a flow‐meter (T106 Transonic Systems). Core temperature was monitored by rectal probe and maintained at 36–37°C by external heating pad (Physitemp, TCAT‐2, Clifton, NJ). Upon completion of all experiments, animals were euthanized by an overdose of the *α*‐chloralose and urethane anesthetic.

### Muscle contraction

The right sciatic nerve was exposed and instrumented with a nerve cuff electrode. The triceps surae muscle group was dissected free and attached to a force transducer (Model MLT1030/D, AD Instruments, Colorado Springs, CO) via the calcaneal tendon. Maximal contractile force (MCF) was determined by stimulation of the triceps surae muscle group with 25, 1 ms impulses delivered at 10 × motor threshold (MT) at a frequency of 100 Hz. The optimal muscle length for tension development was determined by progressively lengthening the muscle and repeating the nerve stimulation until a plateau in tension (peak – baseline) was observed. Rhythmic contractions of the triceps surae muscles were produced at 30% (40 Hz 0.1 ms pulses in 250 ms trains at a rate of 60 trains per min at ~3 × MT) and 60% MCF (40 Hz 0.1 ms pulses in 250 ms trains at a rate of 60 trains per min at ~7 × MT).

### Lumbar sympathetic chain stimulation

A laparotomy was performed and the lumbar sympathetic chain distal to the renal branch of the aorta was dissected free with a blunt glass pipette. A bipolar silver‐wire‐stimulating electrode was attached to the lumbar sympathetic chain between L3 and L4. The electrodes were secured in place and electrically isolated by embedding them in a rapidly curing nontoxic silicone elastomer (Kwiksil, WPI, Sarasota, FL). An isolated constant‐current stimulator (Digitimer DS3, Welwyn City, UK) was used to deliver 1 min of stimulation at frequencies of 2 and 5 Hz (1 ms, 1 mAmp) in random order.

### Experimental procedures

The skeletal muscle vasoconstrictor response evoked by stimulation of the lumbar sympathetic chain at 2 and 5 Hz was determined at rest and during muscle contraction at 30% and 60% of MCF. Sympathetic stimulations were delivered in random order in resting skeletal muscle with sufficient time (~2 min) allowed between stimulations to restore baseline hemodynamic values. Bouts of muscle contraction were 8 min in duration, completed in random order, and separated by 30 min of recovery. During each bout of muscle contraction, stimulations of the lumbar sympathetic chain were delivered 3 and 6 min after the onset of contraction.

Following an additional period of recovery (~20 min), a bolus of BH_4_ (20 mg·kg^−1^) was injected intra‐arterially and followed by continuous intra‐arterial infusion (10 mg·kg^−1^·h^−1^) of BH_4_ by syringe pump for the duration of the experimental protocol. After 20 min of treatment with BH_4_, lumbar sympathetic stimulations were repeated at rest and during skeletal muscle contraction at 30% and 60% of MCF in random order. We have previously demonstrated that muscle force production and the cardiovascular response to sympathetic stimulation are not altered over time when bouts of contraction are repeated in this manner (Jendzjowsky and DeLorey 2013c).

### Effectiveness of BH_4_ supplementation

To assess the effectiveness of BH_4_ supplementation, the vasodilator response to acetylcholine (ACh; 0.1 *μ*g) was measured prior to and following BH_4_ treatment. Small volumes (100 *μ*L) of ACh were injected over ~3 sec in order to avoid flow‐mediated vasodilation. Vehicle injections delivered in this manner did not alter femoral artery blood flow.

The BH_4_ treatment regimen and dosing used in this study is consistent with previous investigations in rats (Gruhn et al. 2001; Noguchi et al. 2010, 2011) and preliminary experiments in our laboratory demonstrated that supplementation performed in this manner improved endothelium‐dependent vasodilation (EDD) and did not alter resting MAP, femoral artery blood flow (FBF), and femoral vascular conductance (FVC).

### Plasma NO_x_ measurement

Venous blood samples (0.6 mL) were collected in EDTA‐containing tubes at rest (*n* = 10) and during the final minute of muscle contraction at 60% MCF (*n* = 10) in Control and BH_4_ treatment conditions. Blood samples were immediately centrifuged at 13,000 g for 15 min at 4°C. The plasma was aliquoted, immediately frozen at −20°C, and subsequently stored at −80°C until analyzed. Plasma samples were defrosted and the concentration of nitrite+nitrate (NO_x_) was measured using a commercially available enzyme‐linked immunoassay kit (Cayman No. 780001, Cayman Chemical Company, Ann Arbor, MI).

### Data analysis

Data were recorded using Chart data acquisition software (AD Instruments). Arterial blood pressure and FBF were sampled at 100 Hz and FVC was calculated as FBF ÷ MAP (mL·min^−1^·mmHg^−1^). Peak force production and fatigue index (([peak force − end‐contraction force] ÷ peak force) × 100) were calculated for each contractile bout. The magnitude of the vasoconstrictor response to sympathetic stimulation was determined by calculation of the mean of the FVC response to sympathetic stimulation (1 min) and expressing it as a percentage change from FVC prior to the stimulation (1 min) in control and BH_4_ conditions. The magnitude of the effect of BH_4_ on sympathetic vasoconstriction at rest and during contraction was determined by expressing the constrictor response in the BH_4_ condition as a percentage of the constrictor response during control conditions. The response to ACh was calculated as the difference between the peak FVC response (~3 sec average) and the preinfusion baseline (~20 sec average) and expressed as a percentage change from the FVC baseline. All data are expressed as mean ± SD.

### Statistics

The effect of BH_4_ supplementation on muscle contractile force, the vasoconstrictor response to sympathetic stimulation, and plasma NO_x_ concentration was analyzed by two‐way repeated measures ANOVA (drug condition x muscle contractile state). Vasodilator responses to ACh were analyzed by one‐way repeated measures ANOVA. When significant F‐ratios were detected, Student–Newman–Keuls post‐hoc testing was completed. A *P*‐value <0.05 was considered statistically significant.

## Results

**Figure 1.**
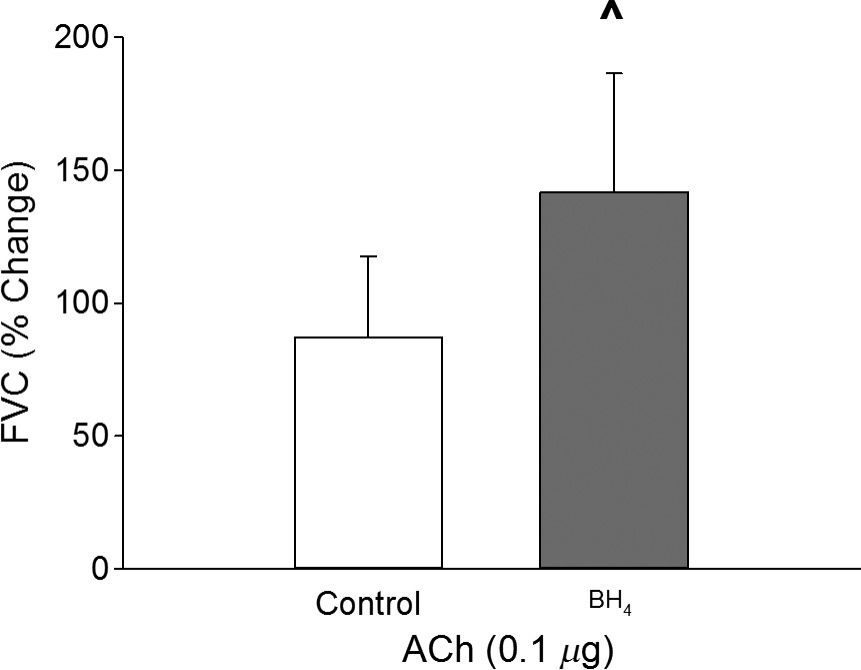
Percentage change of femoral vascular conductance (FVC) in response to bolus injection of acetylcholine (ACh, 0.1 *μ*g) during control conditions (open bars) and following acute BH_4_ supplementation (20 mg·kg^−1^ + 10 mg·kg^−1^·h^−1^, IA shaded bars). Values are mean ± SD. ^ indicates a statistically significant difference from the control condition. A *P*‐value <0.05 was considered statistically significant.

BH_4_ improved (*P* < 0.05) ACh‐mediated vasodilation (Fig. 1). Resting HR was reduced (*P* < 0.05) by BH_4_ supplementation, whereas MAP, FBF, and FVC were not different (*P* > 0.05) in control and BH_4_ conditions (Table 1). Plasma NO_x_ concentration was not different (*P* > 0.05) between control and BH_4_ conditions at rest or during muscle contraction (Table 2).

**Table 1.** Resting hemodynamics

Drug condition	HR (beats·min^−1^)	MAP (mmHg)	FBF (mL·min^−1^)	FVC (mL·min^−1^·mmHg^−1^)
Control	421 ± 35	91 ± 7	3.0 ± 0.7	0.033 ± 0.009
BH_4_	410 ± 11*	90 ± 12	2.6 ± 0.7	0.030 ± 0.007

Heart rate (HR), mean arterial blood pressure (MAP), femoral blood flow (FBF), and femoral vascular conductance (FVC) at rest and during acute tetrahydrobiopterin supplementation (BH_4_, 20 mg·kg^−1^ + 10 mg·kg^−1^·h^−1^, IA). Values are mean ± SD.

* Statistically significant difference from control condition. A *P*‐value <0.05 was considered statistically significant.

**Table 2.** Plasma NO_x_ concentration

Muscle contraction status	Drug condition	NO_*x*_ (*μ*mol/L)
Rest (*n* = 10)	Control	32 ± 16
	BH_4_	28 ± 5
60% MCF (*n* = 10)	Control	27 ± 5
	BH_4_	28 ± 8

Plasma NO_x_ concentration at rest and during contraction at 60% MCF in control conditions and following acute tetrahydrobiopterin supplementation (BH_4_, 20 mg·kg^−1^ + 10 mg·kg^−1^·h^−1^, IA). Values are mean ± SD.

### Effect of BH_4_ on sympathetic vasoconstrictor responses in resting and contracting muscle

**Figure 2.**
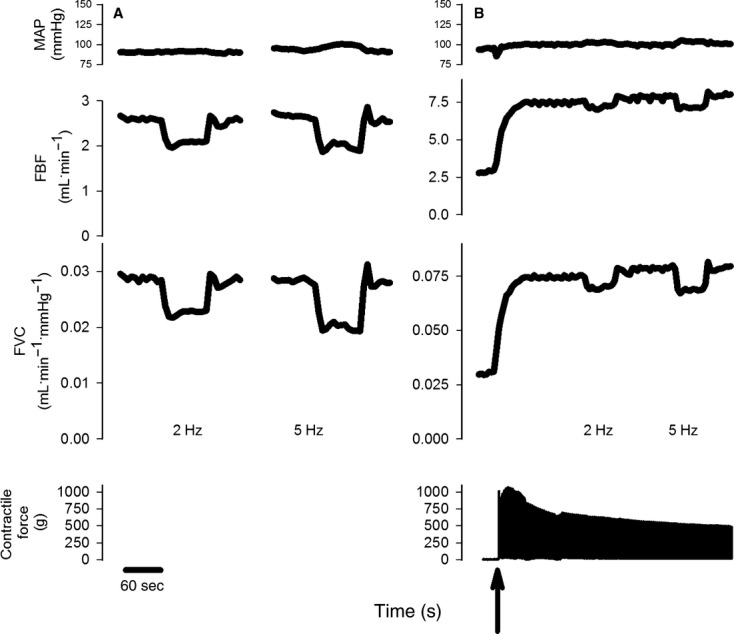
Original data from a representative animal illustrating the response of mean arterial blood pressure (MAP), femoral artery blood flow (FBF), femoral vascular conductance (FVC), and muscle contractile force at rest (Panel A) and during muscle contraction at 60% of maximal contractile force (Panel B). The arrow indicates the onset of contraction. Lumbar sympathetic nerve stimulations were delivered at 2 and 5 Hz in random order at rest and during contraction.

**Figure 3.**
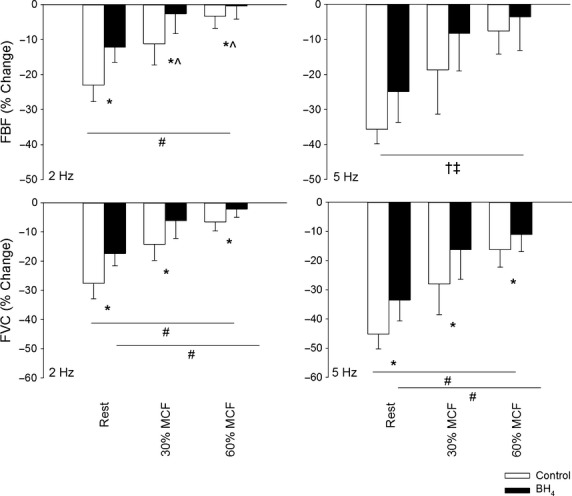
The percentage change of femoral artery blood flow (FBF) and femoral vascular conductance (FVC) in response to sympathetic stimulation at 2 Hz (left) and 5 Hz (right) (left panel) at rest, 30% and 60% maximal contractile force (MCF) during control conditions (open bars) and following BH_4_ supplementation (BH_4_, 20 mg·kg^−1^ + 10 mg·kg^−1^·h^−1^, IA; filled bars). Values are mean ± SD. *indicates a significant difference between drug conditions (significant interaction). ^#^indicates a significant difference between all muscle contractile conditions (significant interaction). ^indicates a significant difference from rest during BH_4_ supplementation. ^†^indicates a significant main effect of BH_4_ supplementation. ^‡^indicates a significant main effect of contractile force (all contractile conditions different). A *P*‐value <0.05 was considered statistically significant.

The response to sympathetic stimulation at rest and during muscle contraction in a representative rat is shown in Figure 2. BH_4_ supplementation reduced (*P* < 0.05) the vasoconstrictor response to sympathetic stimulation delivered at 2 and 5 Hz in resting and contracting skeletal muscle. Compared to the control condition, BH_4_ decreased the constrictor response by 40 ± 10% at the 2 Hz stimulation frequency and 26 ± 12% at the 5 Hz stimulation frequency in resting skeletal muscle (Fig. 3).

Muscle contraction reduced (*P* < 0.05) the vasoconstrictor response to sympathetic stimulation at 2 and 5 Hz in a contraction intensity‐dependent manner in both control and BH_4_ conditions (Table 3 and Fig. 3). During muscle contraction at 30% MCF, BH_4_ treatment decreased the constrictor response to sympathetic stimulation at 2 and 5 Hz by 66 ± 48% and 45 ± 24%, respectively. At 60% MCF, BH_4_ decreased the constrictor response by 64 ± 37% at the 2 Hz stimulation frequency and by 33 ± 17% at the 5 Hz stimulation frequency. The effect of BH_4_ on sympathetic vasoconstriction was greater (*P* < 0.05) in contracting compared to resting muscle, but was not different (*P* > 0.05) between contraction intensities.

**Table 3.** Hemodynamic responses to sympathetic stimulation at rest and during muscle contraction

Stimulation frequency	Contractile state	Drug condition	HR (beats·min^−1^)	MAP (mmHg)	FBF (mL·min^−1^)	FVC (mL·min^−1^·mmHg^−1^)
2 Hz	Rest	Control	−12 ± 10	6 ± 5	−0.7 ± 0.3	−0.0093 ± 0.0031
		BH_4_	−4 ± 5*	6 ± 5	−0.3 ± 0.2*	−0.0053 ± 0.0020*^,^§
	30% MCF	Control	−7 ± 7	3 ± 3†	−0.6 ± 0.4	−0.0089 ± 0.0036
		BH_4_	−4 ± 13*	3 ± 4†	−0.1 ± 0.3*^,^^	−0.0037 ± 0.0045*^,^§
	60% MCF	Control	−5 ± 6†	3 ± 3†	−0.3 ± 0.3^^,^#	−0.0052 ± 0.00323^^,^#
		BH_4_	−1 ± 7*^,^*	2 ± 2†	−0.1 ± 0.3*^,^^	−0.0021 ± 0.0022*^,^§
	Rest	Control	−21 ± 11	16 ± 7	−1.1 ± 0.3	−0.0152 ± 0.0049
		BH_4_	−9 ± 13*	12 ± 7*	−0.6 ± 0.2*	−0.0101 ± 0.0032*
5 Hz	30% MCF	Control	−14 ± 10†	12 ± 4†	−1.1 ± 0.7	−0.0182 ± 0.0078^
		BH_4_	−6 ± 9*^,^†	9 ± 5*^,^†	−0.5 ± 0.7*	−0.0104 ± 0.0080*
	60% MCF	Control	−14 ± 8†	10 ± 4†	−0.6 ± 0.4	−0.0132 ± 0.0047#
		BH_4_	−5 ± 10*^,^†	7 ± 6*^,^†	−0.3 ± 0.6*^,^†^,^‡	−0.0079 ± 0.0035*

Absolute change in heart rate (HR), mean arterial blood pressure (MAP), femoral blood flow (FBF), and femoral vascular conductance (FVC) in response to 2 and 5 Hz sympathetic stimulation at rest and during contraction at 30% and 60% of maximal contractile force (MCF) during control conditions and following acute tetrahydrobiopterin supplementation (BH_4_, 20 mg·kg^−1^ + 10 mg·kg^−1^·h^−1^, IA). Values are mean ± SD.

* Statistically significant main effect of BH_4_.

† Statistically significant main effect of contractile force (different from rest).

‡ Statistically significant main effect of contractile force (different from 30% MCF).

^ Statistically significant difference from rest within specified drug condition (significant interaction).

# Statistically significant difference from 30% MCF within specified drug condition (significant interaction).

§ Statistically significant difference between all contractile states within specified drug condition (significant interaction). A *P*‐value <0.05 was considered statistically significant.

### Muscle force production and exercise hyperemia

Muscle force production was not different (*P* > 0.05) between control and BH_4_ conditions at 30% MCF (Control: 587 ± 132 g; BH4: 561 ± 79 g) and 60% MCF (Control: 983 ± 147 g; BH4: 935 ± 157 g). Fatigue index was also not different (*P* > 0.05) between control (30% MCF: 35 ± 15%; 60% MCF: 43 ± 12%) and BH_4_ conditions (30% MCF: 33 ± 12%; 60% MCF: 50 ± 13%). The hemodynamic response to muscle contraction at 30% and 60% MCF was not different (*P* > 0.05) between control and BH_4_ conditions (Table 4).

**Table 4.** Hemodynamic response to muscle contraction

Muscle contraction	Drug condition	HR (beats·min^−1^)	MAP (mmHg)	FBF (mL·min^−1^)	FVC (mL·min^−1^·mmHg^−1^)
30%	Control	10 ± 8	4 ± 6	3.1 ± 0.7	0.033 ± 0.009
	BH_4_	9 ± 13	3 ± 8	3.1 ± 1.1	0.035 ± 0.015
60%	Control	10 ± 6	6 ± 5	4.8 ± 0.9*	0.050 ± 0.010*
	BH_4_	8 ± 14	4 ± 4	4.3 ± 1.4*	0.047 ± 0.015*

Absolute increase of heart rate (HR), mean arterial pressure (MAP), femoral artery blood flow (FBF), and femoral vascular conductance (FVC) in response to muscle contraction at 30% and 60% of maximal contractile force during control conditions and following acute tetrahydrobiopterin supplementation (BH_4_, 20 mg·kg^−1^ + 10 mg·kg^−1^·h^−1^, IA). Values are mean ± SD.

* Significant main effect of contractile force (60% >30% MCF). A *P*‐value <0.05 was considered statistically significant.

## Discussion

The purpose of this investigation was to determine whether acute BH_4_ supplementation would blunt sympathetic vasoconstrictor responsiveness in resting and contracting skeletal muscle. Consistent with our hypothesis, sympathetic vasoconstrictor responsiveness was diminished in resting and contracting skeletal muscle following acute treatment with BH_4_ in this study.

BH_4_ is an essential cofactor for NO production (Forstermann 2010; Forstermann and Sessa 2012) and is required for normal enzymatic function of all NOS isoforms (Katusic and d'Uscio 2004; Katusic et al. 2009). BH_4_ acts as an allosteric enzyme that stabilizes the NOS complex and prevents uncoupling of NOS enzyme activity. Increased BH_4_ availability may provide additional substrate for NOS enzymes or reduce the uncoupling of eNOS (Vasquez‐Vivar et al. 2003; Bevers et al. 2006) and nNOS (Heinzel et al. 1992; Pou et al. 1992; Sun et al. 2008) which limits superoxide (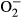 ) production, leads to de novo synthesis of NO, and increases NO bioavailability (Bevers et al. 2006; Forstermann 2010; Forstermann and Sessa 2012; Santhanam et al. 2012). The dose of BH_4_ utilized in this study has been shown to increase circulating BH_4_ concentrations and BH_4_ content in skeletal muscle and isolated arteries (Gruhn et al. 2001; Delp et al. 2008; Noguchi et al. 2010, 2011). Functionally, BH_4_ supplementation has been shown to increase flow‐mediated NO production in isolated arteries (Sindler et al. 2009) and improve ACh‐ and flow‐mediated vasodilation in adults with normal (Higashi et al. 2002) and reduced endothelial function (Higashi et al. 2002; Eskurza et al. 2005; Cosentino et al. 2008). Supplementation with BH_4_ has also been shown to increase vascular compliance in older men (Pierce et al. 2012). Collectively, these studies indicate that acute BH_4_ supplementation improves NO bioavailability and NO‐mediated vascular function. Consistent with previous studies, acute treatment with BH_4_ improved ACh‐mediated vasodilation in this study. Despite improved ACh‐mediated vasodilation, BH_4_ supplementation did not result in an increase in plasma NO_x_ at rest or during muscular contraction. The lack of an increase in plasma NOx is difficult to reconcile; however, it is possible that BH_4_ supplementation improved tissue NOx content and not circulating NOx levels. Consistent with the present findings, supplementation with the NO precursor arginine had no effect on plasma NO_x_ in humans and sedentary rats (Xiao et al. 2003; Forbes and Bell 2011; Forbes et al. 2013).

Sympathetic vasoconstrictor responsiveness was reduced following treatment with BH_4_ in this study, suggesting that supplementation with BH_4_ augmented NO‐mediated inhibition of sympathetic vasoconstriction. Our laboratory and others have demonstrated that NO inhibits sympathetic vasoconstriction in resting and contracting skeletal muscle (Habler et al. 1997; Thomas and Victor 1998; Chavoshan et al. 2002; Donato et al. 2007; Behnke et al. 2011; Jendzjowsky and DeLorey 2013c). Indeed, NO derived from both eNOS and nNOS has been shown to inhibit sympathetic vasoconstriction in resting and contracting skeletal muscle (Ohyanagi et al. 1992; Habler et al. 1997; Thomas and Victor 1998; Thomas et al. 1998, 2003; Jendzjowsky and DeLorey 2013c). In resting skeletal muscle, NO‐mediated inhibition of sympathetic vasoconstriction appears to be predominately mediated by NO derived from eNOS (Grange et al. 2001; Jendzjowsky and DeLorey 2013b). It is well established that BH_4_ treatment reduces eNOS uncoupling and improves eNOS‐mediated vascular function (Katusic and d'Uscio 2004; Katusic et al. 2009). Thus, the increased blunting of sympathetic vasoconstriction in resting skeletal muscle appears consistent with improved eNOS function following BH_4_ supplementation. The augmented inhibition of sympathetic vasoconstriction following BH_4_ supplementation suggests that eNOS activity could be a therapeutic target to blunt sympathetic vasoconstriction and reduce vascular resistance. This may be particularly effective in conditions where nNOS expression is reduced, such as muscular dystrophy, heart failure, etc. (Thomas et al. 1998, 2003; Sander et al. 2000; Vongpatanasin et al. 2011; Notarius et al. 2014).

During muscular contraction, increased endothelial shear stress and elevated intracellular [Ca^++^] lead to greater eNOS and nNOS‐mediated NO production. In this study, the blunting of sympathetic vasoconstrictor responsiveness during BH_4_ treatment was greater in contracting compared to resting skeletal muscle, suggesting that treatment with BH_4_ may be particularly effective at increasing “stimulated” NO production. nNOS‐derived NO appears to be particularly important for the inhibition of sympathetic vasoconstriction in contracting skeletal muscle (Thomas et al. 1998, 2003; Lau et al. 2000; Grange et al. 2001). The effects of BH_4_ on nNOS‐mediated vascular function are not well established and have received relatively little attention in the scientific literature. nNOS is primarily localized in the muscle sarcolemma and treatment with BH_4_ has been shown to increase BH_4_ content in skeletal muscle cells (Harding et al. 2004). Thus, increased BH_4_ availability may facilitate increased nNOS‐mediated NO production in contracting muscle. However, the increased shear stress will increase eNOS‐mediated NO production during exercise and may upregulate eNOS‐mediated sympatholysis. Our laboratory recently reported that the contribution of NO derived from eNOS and nNOS to the inhibition of sympathetic vasoconstriction were proportional during skeletal muscle contraction (Jendzjowsky and DeLorey 2013b). This finding indicates that eNOS‐derived NO is an important contributor to sympatholysis in healthy rats and suggests that BH_4_ supplementation may upregulate eNOS‐mediated sympatholysis. Further investigation is required to determine the specific NOS isoform responsible or the relative contribution of each isoform for the improved blunting of sympathetic vasoconstriction following BH_4_ treatment. However, regardless of the specific isoform responsible, the greater blunting of sympathetic vasoconstriction in contracting compared to resting muscle suggests that increased BH_4_ availability may be particularly effective for “stimulated” NO production and that BH_4_ supplementation may improve skeletal muscle vascular control in conditions where sympatholytic capacity may be diminished (e.g. aging, heart failure, etc.) (Lang et al. 1997; Dinenno et al. 2005; Vongpatanasin et al. 2011; Mortensen et al. 2012; Notarius et al. 2014).

BH_4_ may have also reduced sympathetic vasoconstrictor responsiveness through its antioxidant properties. We and others (Zhao et al. 2006; Jendzjowsky and DeLorey 2013a) have shown that treatment with a superoxide scavenger reduces sympathetic vasoconstrictor responsiveness in resting and contracting skeletal muscle. In vitro, BH_4_ has been shown to scavenge 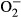 , thiol radicals, and peroxynitrite (Heales et al. 1988; Gramsbergen et al. 2002; Kuzkaya et al. 2003; Katusic et al. 2009). However, in vivo, BH_4_ does not appear to contribute to the scavenging of 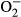 and treatment with tetrahydroneopterin, a BH_4_ analogue that has antioxidant properties but does not alter NOS function, did not improve ACh‐mediated vasodilation in the forearm of smokers with reduced endothelial function (Heitzer et al. 2000; Vasquez‐Vivar et al. 2002).

### Effect of BH_4_ treatment on skeletal muscle blood flow

BH_4_ reduced resting HR but did not alter resting MAP, FBF, and FVC, consistent with a previous study that reported no effect of BH_4_ on resting limb blood flow (Eskurza et al. 2005). The limb blood flow response (increase in FBF and FVC) to muscle contraction was also not altered by treatment with BH_4_, despite enhanced EDD and greater inhibition of sympathetic vasoconstriction in this study. These findings appear contradictory; however, multiple signaling pathways regulate the hyperemic response to contraction in an integrative and redundant manner and blockade of and/or adaptations in an individual signaling pathway often do not impact the overall blood flow response to exercise (Laughlin and Korzick 2001). Indeed, to our knowledge, it has not been demonstrated that an improvement in EDD consistently results in an augmented hyperemic response to exercise. Moreover, the contribution of NO to the regulation of bulk limb blood flow in response to muscle contraction remains controversial (Clifford and Hellsten 2004). Previous studies have reported that NO was not required for a “normal” hyperemic response to exercise (Radegran and Saltin 1999; Frandsenn et al. 2001), whereas others have demonstrated that exercise hyperemia was reduced following NOS inhibition (Hirai et al. 1994; Dietz et al. 1997). Recent evidence suggests that NO derived from nNOS may be involved in the distribution of muscle blood flow during exercise (Copp et al. 2013). It is well recognized that functional sympatholysis is necessary to oppose the increased sympathetic drive during exercise and contributes to the regulation of muscle blood flow and systemic blood pressure (Buckwalter and Clifford 2001; Delp and O'Leary 2004; Thomas and Segal 2004). However, it has been shown that sympatholysis may facilitate the distribution of blood flow between and within muscles and not alter bulk limb blood flow during contraction (VanTeeffelen and Segal 2003). Consistent with this notion, our laboratory has recently reported that short‐term exercise training enhanced NO‐mediated inhibition of evoked sympathetic vasoconstriction, while the limb blood flow response to muscle contraction was not altered (Jendzjowsky and DeLorey 2013c). Thus, in this study, it is conceivable that the BH_4_‐mediated improvements in EDD and inhibition of sympathetic vasoconstriction may have enhanced the distribution of blood flow between and within muscles at rest and during contraction.

### Experimental considerations and limitations

An additional experimental condition where NO production was inhibited following BH_4_ treatment may have provided complementary evidence to the increase in EDD that the effects of BH_4_ on sympathetic vasoconstriction were mediated by an NO‐dependent mechanism. However, a NOS blockade condition would require a total of six bouts of muscle contraction in each rat. Preliminary experiments in our laboratory involving six bouts of contraction and recovery did not yield reproducible levels of muscle force production and constrictor responses to sympathetic stimulation during the final two bouts of contraction and therefore a NOS inhibition condition was not feasible.

BH_4_ is also involved in the synthesis of norepinephrine (NE) in a reaction catalyzed by the enzyme tyrosine hydroxylase (Nagatsu 1983). In the cutaneous vascular bed, BH_4_ supplementation has been shown to augment cold induced vasoconstriction without altering postsynaptic receptor function suggesting that the increased vasoconstriction was mediated by increased NE production (Lang et al. 2009, 2010). Our finding of reduced sympathetic vasoconstrictor responsiveness suggests that an increase in NE production following treatment with BH_4_ was unlikely in this study.

## Conclusions

The current data demonstrate that acute BH_4_ supplementation reduced sympathetic vasoconstrictor responsiveness in resting and contracting skeletal muscle. These findings in healthy rats suggest that BH_4_ bioavailability may be an important factor in the inhibition of sympathetic vasoconstriction and that BH_4_ supplementation may be an appropriate therapy in conditions characterized by elevated sympathetic vascular resistance and diminished sympatholysis. Further investigation is required to identify the cellular mechanism by which BH_4_ supplementation reduces sympathetic vasoconstrictor responsiveness.

## Conflict of Interest

The authors have no conflict of interest to disclose.
